# Coin-shaped corneal endothelial scar in herpes zoster ophthalmicus: a case report

**DOI:** 10.1186/s13256-022-03319-5

**Published:** 2022-03-17

**Authors:** Seung Hyeun Lee, Yeoun Sook Chun, Kyoung Woo Kim

**Affiliations:** Department of Ophthalmology, Chung-Ang University Hospital, Chung-Ang University College of Medicine, 102 Heukseok-ro, Dongjak-gu, Seoul, 06973 Republic of Korea

**Keywords:** Anterior uveitis, Coin-shaped corneal endothelial scar, Facial herpes zoster, Herpes zoster ophthalmicus, Herpes zoster virus, Recurrence

## Abstract

**Background:**

Herpes zoster ophthalmicus includes a wide spectrum of lesions at the ocular surface, including epithelial, stromal, endothelial keratitis, and uveitis. Thus far, the occurrence of corneal endothelial disorder in herpes zoster ophthalmicus and the causative virus have not been confirmed, and the differential diagnosis and establishment of therapeutic strategies are challenging. Corneal endothelial coin-shaped lesions are well known to occur in cytomegalovirus-related corneal endotheliitis but have not been reported in patients with herpes zoster ophthalmicus.

**Case presentation:**

A 39-year-old Asian female was referred to our ophthalmology department with recurrent anterior uveitis accompanied by coin-shaped corneal endothelial scar-like lesions that appeared after right facial herpes zoster. Diffuse corneal stromal haziness was mostly limited in the anterior stroma. The coin-shaped corneal endothelial lesions were separate from stromal lesions and showed a high-reflective scar-like line in sections of anterior segment optical coherence tomography. Anterior uveitis recurred each time she discontinued oral antiviral drug treatment for 12 months after the first event, but was remitted by the maintenance medications of combined topical ganciclovir gel with oral valaciclovir, at a dose lower than the usual adult dose, for acute or recurrent zoster-associated anterior uveitis. Corneal endothelial function remained normal and corneal endothelial and stromal lesions were unchanged throughout the treatment and follow-up period.

**Conclusions:**

In patients with a history of facial herpes zoster with coin-shaped corneal endothelial scar accompanying recurrent anterior uveitis, suspicion for active varicella-zoster virus is warranted, and prolonged intake of oral antiviral agents is required despite varicella-zoster virus DNA not being detected in aqueous humor.

## Background

Herpes zoster ophthalmicus (HZO) is a disease of reactivation and repopulation of varicella-zoster virus (VZV) throughout the ophthalmic branch (V1) from the trigeminal nerve. Once VZV invades the nasociliary nerve, in particular, various eye tissues such as conjunctiva, cornea, sclera, iris, choroid, optic nerve, and retina are affected and become inflamed [1]. Corneal complications occur in about 65–75% of HZO, and in order of the frequency they include punctate epithelial keratitis, pseudodendritic keratitis, anterior stromal keratitis, keratouveitis, neurotrophic keratitis, and disciform stromal keratitis [2, 3]. Although most of these corneal complications show a transient course, limited cases may progress to a chronic form, such as neovascularized lipid keratopathy exclusively in nummular anterior stromal keratitis and disciform stromal keratitis [1].

We first present a case of coin-shaped corneal endothelial scar in HZO that mimicked cytomegalovirus (CMV)-induced coin-shaped corneal endotheliitis and was remitted by combination therapy of low-dose oral valacyclovir and topical ganciclovir gel.

## Case presentation

A 39-year-old Asian female was referred for corneal endothelial abnormality with recurrent anterior uveitis and intraocular pressure (IOP) elevation in her right eye. One month before her initial ocular symptoms, she had skin lesions with pain, having noticed blisters on the forehead and swelling with redness on her right upper and lower eyelids; she had been diagnosed with zoster at the right ophthalmic branch dermatome of the trigeminal nerve. As a usual treatment of zoster, the initial episode of the zoster was treated with oral famciclovir 250 mg three times daily for 1 week. She was previously diagnosed with hepatitis, but she had no other past medical, surgical, social, or family history to specify.

**Fig. 1:**
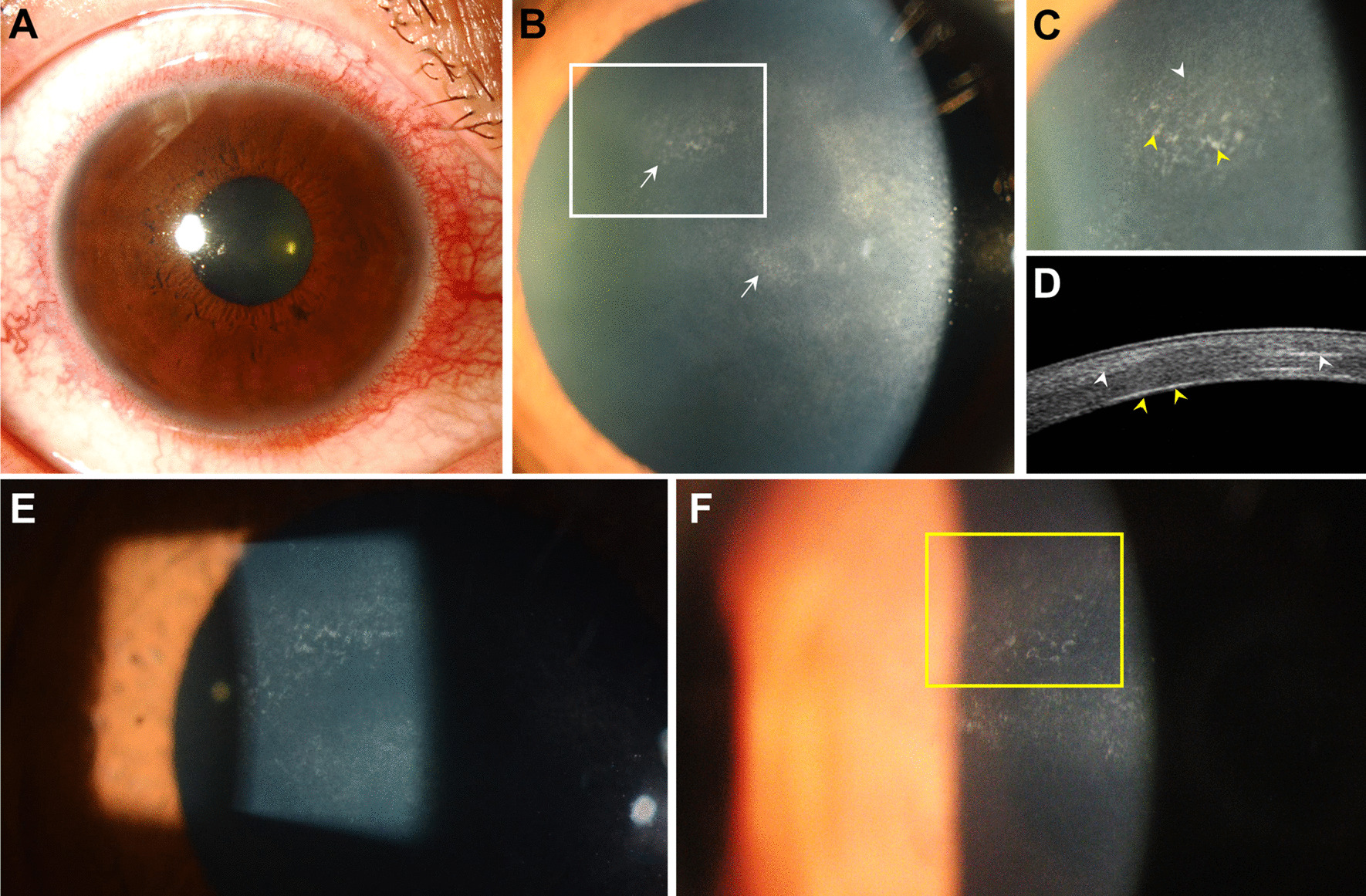
The coin-shaped corneal endothelial scar with inflammation at the initial referral. **A**, **B** Ciliary injection (**A**) and multiple corneal hazy lesions (**B**, arrows) are shown. **C**, **D** At 10 o’clock mid-periphery of the cornea (**C**, the magnified view of the white rectangle in **B**), a coin-shaped corneal endothelial scar-like lesion comprising scar-like opaque foci (**C**, yellow arrowheads) with overlying corneal stromal haziness (**C**, white arrowhead) is noted. Anterior segment optical tomography (**D**) at the area of the lesion (**C**), accordingly, revealed a highly reflective line at the level of Descemet’s membrane (**D**, yellow arrowheads) and overlying stromal opaque lesions (**D**, white arrowheads). **E** After 5 months, the coin-shaped endothelial lesions remained without noticeable change. **F** At 1 year, the coin-shaped corneal endothelial scar-like lesions became sparse (inside the yellow rectangle)

On her first visit to our institute, the corrected visual acuity of the right eye was 20/20 and IOP was 14 mmHg. Copious ciliary injection was noted (Fig. 1A), and at 10 o’clock mid-periphery of the cornea and the center, coin-shaped corneal endothelial scar-like lesions were observed between or below the areas of cornea stromal haziness (Fig. 1B, C). On anterior segment ocular optical tomography, corneal endothelial lesions appeared as highly reflective lines at the level of Descemet’s membrane (Fig. 1D). There was no corneal edema in the area of corneal endothelial lesions, and the corneal endothelial cell density was normal.

Herpes simplex virus (HSV), CMV, VZV, Epstein–Barr virus (EBV), and human herpesvirus (HHV) type 6 were all negative by multiplex polymerase chain reaction (PCR) from aqueous humor sample. Serological anti-human immunodeficiency virus antibody, antinuclear antibody, rheumatoid factor, and human leukocyte antigen-B51 were negative. Differential blood cell counts were also within the normal range. However, based on the history of typical facial zoster at V1 dermatome and the concurrent recurrent anterior uveitis, the patient was presumed to have HZO, and oral valaciclovir (ValACV) 500 mg twice per day, topical ganciclovir gel (Virgan, Laboratiores Théa, Clermont-Ferrand, France) five times per day, and 0.1% fluorometholone four times per day were started. After 5 months, while anterior uveitis was in remission, coin-shaped endothelial lesions remained similar in appearance (Fig. 1E). Although the anterior uveitis recurred once within 10 days after discontinuance of ValACV, there was no additional recurrence of anterior uveitis with intake of oral ValACV 500 mg twice per day for 1 year afterward. At 1 year, the coin-shaped corneal endothelial scar-like lesions remained but became sparse (Fig. 1F). The corneal endothelial cell density was normal, without occurrence of corneal edema.

## Discussion and conclusion

One-third of HZO patients experience attacks of anterior uveitis that occur 2–4 weeks after the initial onset of HZO, and they also accompany corneal epithelial, stromal, or endothelial abnormalities [4]. The diagnosis of zoster anterior uveitis is usually clinically based on the history of herpes zoster, and as a confirmatory test, a polymerase chain reaction test is performed using aqueous humor. Despite the ability of PCR to distinguish viruses such as HSV, VZV, and CMV, the sensitivity of the test may decrease during the administration of antiviral agents, as in this case [4]. In addition, while the multiplex PCR performed in this case has the advantage of detecting various targets at once, false negative results may occur due to competition and interference between primers [5, 6].

The chronic or recurrent course of HZO usually occurs in the form of anterior uveitis, whereas corneal complications usually present epithelial keratitis or pseudodendritic keratitis early in the disease. Although relatively rare, other known corneal involvements of HZO include lipid keratopathy caused by disciform stromal keratitis, corneal thinning, corneal perforation, and persistent corneal edema caused by severe endotheliitis [1, 3, 7]. However, the coin-shaped corneal endothelial lesion observed in this case has not been reported. It is presumed to be a scar lesion because its shape did not change despite the clinical improvement observed on anterior segment ocular optical tomography. According to a previous study that reported detecting VZV DNA on the ocular surface for more than 1 month after onset of HZO [8], it is plausible that VZV continues damaging corneal tissue during the prolonged period. Also, in this case, VZV might cause both severe stromal keratitis and corneal endotheliitis, which might have caused coin-shaped inflammatory scar over recurrences.

The 50% effective dose (ED50) of the expected biological effects of acyclovir (ACV) against VZV is known to be between 2.06 and 6.28 μM [9]. The concentration of ACV in the anterior chamber reached 3.26 μM when oral acyclovir 400 mg was taken five times daily [10]. Based on the similarity of blood ACV concentrations between 4000 mg daily oral ACV and 1000 mg daily oral ValACV [11], it can be assumed that the ACV concentration of about 6–7 μM dose was maintained in the anterior chamber after oral 500 mg of ValACV twice daily. The four to six times daily use of topical 3% ACV ointment, which is known as the effect equivalent of 0.15% ganciclovir [12], causes concentrations of up to 7.5 μM in the anterior chamber [13] and it is even higher than ED50 of ACV to VZV. In our case, the remission of anterior uveitis, in this case, was oral antiviral dependent. This is consistent with a previous study showing that local preparations were ineffective in early HZO, which may be due to VZV pathologically invading the nerves and blood vessels behind the eye; thus, oral antiviral agents may affect the tissue and trigeminal nerve pathways [14]. Furthermore, the recurrence after discontinuing oral ValACV and the remission after reintake of oral ValACV suggests that this case is associated with the reactivation of VZV.

Despite the corneal endothelial lesion, in this case, not resembling the coin-shaped lesion usually observed in CMV corneal endotheliitis, we suggest that severe stromal inflammation in HZO may accompany adjacent round endothelial scar at deeper layers of the endothelium.

In conclusion, in patients with a history of facial herpes zoster with coin-shaped corneal endothelial scar accompanying recurrent anterior uveitis, suspicion of active VZV is warranted, and prolonged intake of oral antiviral agents is required, despite VZV DNA not being detected in aqueous humor.

## Data Availability

All data generated or analyzed during this study are included in this published article.

## Abbreviations

HZO: Herpes zoster ophthalmicus
HSV: Herpes simplex virus
CMV: Cytomegalovirus
VZV: Varicella zoster virus
EBV: Epstein–Barr virus
HHV: Human herpes virus
IOP: Intraocular pressure
ACV: Acyclovir
